# Role of circulating exosomal biomarkers and their diagnostic accuracy in pancreatic cancer

**DOI:** 10.1002/jgh3.12848

**Published:** 2022-11-25

**Authors:** Menazir Sha, Basir Kunduzi, Saied Froghi, Alberto Quaglia, Brian Davidson, Giuseppe K Fusai

**Affiliations:** ^1^ University College London, Medical School London UK; ^2^ Division of Surgery and Interventional Sciences/UCL Royal Free Hospital London UK; ^3^ Renal Transplant Unit Guy's Hospital London UK; ^4^ Department of HPB and Liver Transplantation Royal Free Hospital London UK; ^5^ Department of Cellular Pathology Royal Free Hospital London UK

**Keywords:** cancer biomarker, exosomal biomarker, pancreatic cancer

## Abstract

**Background and Aim:**

New biomarkers have the potential to facilitate early diagnosis of pancreatic cancer (PC). Circulating exosomes are cell‐derived protein complexes containing RNA that can be used as indicators of cancer development. The aim of this review is to evaluate the current literature involving PC patient groups for highly accurate exosomal biomarkers.

**Methods:**

The literature search followed Preferred Reporting Items for Systematic Reviews and Meta‐Analyses guidelines. Eight‐hundred and seventy‐five studies were identified across various databases (Ovid MEDLINE, Embase, and Cochrane) published between 2009 and 2020. Nine studies fulfilled the inclusion criteria: human PC patients, diagnosis as outcome of interest, serum biomarker of exosomal content, reporting of diagnostic values, and disease progress. Area under the curve (AUC) of the exosomal biomarker was compared against that of CA19‐9.

**Results:**

Nine papers were reviewed for relevant outcomes based on the inclusion criteria. These studies involved 565 participants (331 PC, 234 controls; male/female ratio 1.21; mean age 64.1). Tumor staging was reported in all studies, with 45.6% of PC patients diagnosed with early‐stage PC (T1–2). The mRNA panel (ARG1, CD63, CK18, Erbb3, GAPDH, H3F3A, KRAS, ODC1) and GPC 1 reported the highest performing sensitivity and specificity at 100% each. The microRNA panel (miR‐10b, miR‐21, miR‐30c, miR‐181a, and miR‐let7a), mRNA panel (ARG1, CD63, CK18, Erbb3, GAPDH, H3F3A, KRAS, ODC1), and GPC 1 showed a perfect AUC of 1.0. Five studies compared the AUC of the exosomal biomarker against CA19‐9, each being superior to that of CA19‐9.

**Conclusion:**

The potential of exosomal biomarkers remains promising in PC diagnosis. Standardization of future studies will allow for larger comparative analyses and overcoming contrasting findings.

## Graphical abstract

This systematic review aims to evaluate the current literature involving pancreatic cancer patient groups in search of highly accurate exosomal biomarkers. We reviewed 9 studies which involved 565 participants (331 PC and 234 controls). The mRNA panel (ARG1, CD63, CK18, Erbb3, GAPDH, H3F3A, KRAS, and ODC1) and GPC1 reported the highest performing sensitivity and specificity at 100% each.

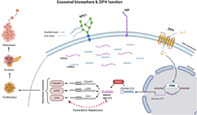

## Introduction

Pancreatic cancer (PC) has the fourth highest mortality in relation to cancer in Europe.^1^ Although new treatment for many cancers has improved survival, novel interventions have had a minimal impact on PC survival. The median survival for PC patients is 18 months, with less than 10% surviving to 5 years.^1^, ^2^ Only a minority of patients at presentation can undergo a potentially curative resection,^3^ and the majority of resected patients die of recurrence. Prognostic outcomes may greatly improve if there is a shift from patients being diagnosed from late stage to an early or precancerous stage.^4^, ^5^ Unfortunately, there has been little progress in the research on potential diagnostic tools that enable earlier diagnosis.^6^ Current strategies for diagnosis include various radiological imaging techniques along with endoscopic retrograde cholangiopancreatography (ERCP) and biopsies among other common practices for tumor confirmation. Unfortunately, radiological imaging alone is often inadequate because it has only moderate sensitivity and specificity for detecting PC. In addition, early pancreatic lesions are of particular importance, and these are often missed by these modalities completely.^7^ Newer studies have looked into the role of artificial intelligence and deep learning methods in diagnosing PC mainly hinging on detecting early CT changes.^8^, ^9^ Screening of “at‐risk” individuals has been proposed as the optimal solution. Although at‐risk patients can be identified, there is currently no clinically established strategy for screening these patients.^6^, ^10^

Carbohydrate antigen 19‐9 (CA19‐9) is presently the only biomarker for PC in clinical use. CA19‐9 is principally used to measure therapeutic response and to determine prognosis, although it has also frequently been used in the diagnosis of PC.^11^, ^12^ However, there are some challenges to the use of CA19‐9 as a screening tool. First, it has been reported to have sub‐optimal sensitivity (41–86%) and specificity (33–100%).^13^, ^14^ Within an asymptomatic population, mass screening is rather ineffective because of its poor positive predictive value.^15^, ^16^ Also, only 65% of early‐stage pancreatic ductal adenocarcinoma (PDAC) patients present with elevated CA19‐9 in sera.^17^ CA19‐9 has frequently been demonstrated to be elevated in various other gastrointestinal malignancies as well as benign conditions such as pancreatitis.^18^, ^19^ Furthermore, CA19‐9 is not expressed by around 5–10% of the Caucasian population who possess a Lewis‐negative genotype.^20^ Thus, it is vital to explore novel biomarkers that may be more effective in the diagnosis of PC.

Circulating biological markers have been proposed as a potential screening solution for PC patients. Serum biomarkers, in particular, hold great clinical potential, as they can be obtained noninvasively in comparison to tissue markers. These biomarkers can be difficult to detect, as they are diluted by the various other components of blood.^21^, ^22^ However, recent technological advances in gene‐expression microarrays, proteomics, and immunology have facilitated biomarker research.^23^

Numerous biomarkers exist, ranging from inflammatory markers and metabolites to gene sequences and circulating tumor cells.^24^ Of all the potential biomarkers, exosomes (extracellular vesicles) in particular present a promising candidate for diagnostics in relation to cancer.

**Figure 1:**
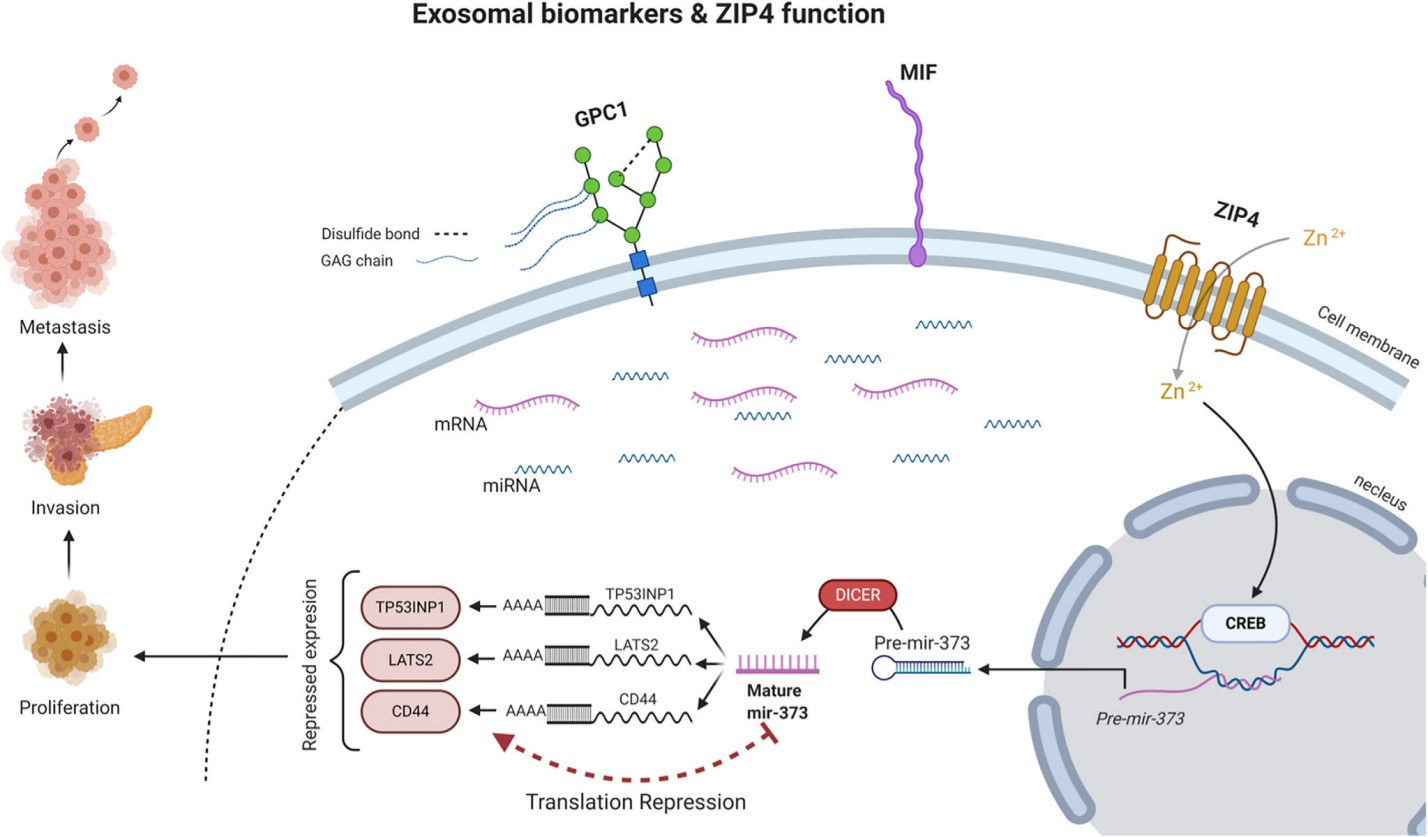
Membrane‐bound surface proteins (GPC 1, MIF, and ZIP 4) have been found to be upregulated in pancreatic cancer‐derived exosomes. ZIP 4, an ion transporter, regulates intracellular zinc homeostasis. The differential expression of ZIP 4 has been reported in pancreatic cancer.

Exosomes are extracellular vesicles that are released by almost every type of cell, including cancer cells (Fig. 1).^25^, ^26^ These vesicles contain a variety of intracellular contents such as DNA, mRNA, miRNA, proteins, and metabolites.^27^ Studies support the theory that they are mediators of intercellular signaling that dynamically respond to the different stressors of the secreting cell microenvironment (implying further specificity).^28^

They are important in normal physiologic processes, such as immune response, lactation, and neuronal function, but emerging evidence has also found that exosomes are involved in the development of numerous pathologies such as neurodegenerative diseases and cancer.^29^

Every cell in the body releases exosomes including cancer cells.^30^ Exosomes from cancer cells facilitate communication with other cells. Cancer cells have also been shown to secrete exosomes at a higher rate than normal cells.^31^ Exosomes show high levels of heterogeneity that are derivative of the cell that produces them.^32^ Specifically within PC, genomic pleomorphism is abundant with known mutations (KRAS, BRCA) and other more novel ones associated with chromatin modification, DNA damage repair, etc.^33^ Thus, exosomes potentially contain numerous specific markers that can be identified as diagnostic of PC.^29^, ^33^

Exosomes are particularly novel as biomarkers because just a decade ago their detection was considered too difficult and ineffective. Recent technological advances in immunoassay procedures, for example, have helped develop new, effective, timely, and precise mechanisms to identify exosomes and analyze their molecular contents.^34^ This, coupled with an increase in our etiological understanding of cancer and the function of exosomes, has led to an increased focus on the potential diagnostic capability of circulating exosomes in cancer.^35^, ^36^, ^37^

The aim of this review is to evaluate the current literature involving PC patient groups for highly accurate exosomal biomarkers, highlight comparisons with CA19‐9 where reported, and identify performance discrepancies between studies.

## Methods

### Search strategy

A literature search was performed by M.S., S.F. on November 2020 using the MeSH/EMTREE terms “pancreas,” “cancer,” “diagnosis,” “detection,” “exosome,” “biomarker,” and “marker” as keywords in a Boolean search on the electronic databases of Ovid MEDLINE(R), Embase, and Cochrane Central Register of Controlled Trials. The search was limited to full‐text articles available in English. Our review focused on identifying observational studies. Also, in concordance with the “novel” aspect of the review, only publications from the last 10 years were included. Duplicates were then removed with the use of citation software. An additional search using Google Scholar was performed to ensure discovery of all suitable studies.

### Study selection criteria

Titles and abstracts of all studies retrieved from the literature search were reviewed by two authors (M.S. and S.F.) to determine relevance.

#### Inclusion criteria

Studies were included according to the following criteria: (1) Population of interest were human PC patients; (2) Outcome of interest involved diagnosis of the cancer; (3) Biomarker assessed was that of an exosome or exosomal content; (4) Biomarkers were accessible in serum (noninvasively); (5) Diagnostic performance values (the ability to distinguish between PC and controls) of biomarkers were included: specifically, sensitivity, specificity, and area under the curve (AUC); (6) Disease progress, such as tumor staging, was reported to establish diagnostic performance in different stages of disease.

#### Exclusion criteria

Studies were excluded according to the following criteria: (1) Publications in the form of letters, case reports, editorials, or reviews; (2) Studies assessing only animal models; (3) Studies only evaluating prognostic and/or predictive element(s) of biomarkers; (4) Studies with no control group; (5) Studies which primarily assessed biomarker detection method rather than their diagnostic capability.

### Quality assessment

Two reviewers (M.S., S.F.) assessed quality of eligible studies via the Grading of Recommendations Assessment, Development and Evaluation (GRADE) quality of evidence tool, recommended by the Cochrane Collaborative Group.^38^ Studies were scored as high, moderate, low, or very low. Only studies determined to be of “moderate” to “high” quality via the GRADE tool were preserved for inclusion in the review. Consensus using the author coefficient of agreement was achieved to resolve differences between reviewers.

### Data collection and handling

This review was prospectively registered on PROSPERO.^39^ We report our findings according to the Preferred Reporting Items for Systematic Reviews and Meta‐Analyses statements (PRISMA) guidelines.^40^ Data were extracted from all included studies and collated for the parameters of interests. Study characteristics that were extracted included biomarker(s) examined, detection method, number of patients, mean age, gender ratio, diagnostic performance of biomarker(s), predictive values, and any comments regarding the study. Sensitivity, specificity, and AUC values of biomarkers were compared. Where individual biomarkers were repeated between studies, their diagnostic accuracies were compared for any differences. Where reported, the AUC values of CA19‐9 were compared to the exosomal biomarker assessed in the respective study. Statistical analyses were conducted using Graph‐Pad Prism 8 (GraphPad Software, CA, USA).

## Results

**Figure 2:**
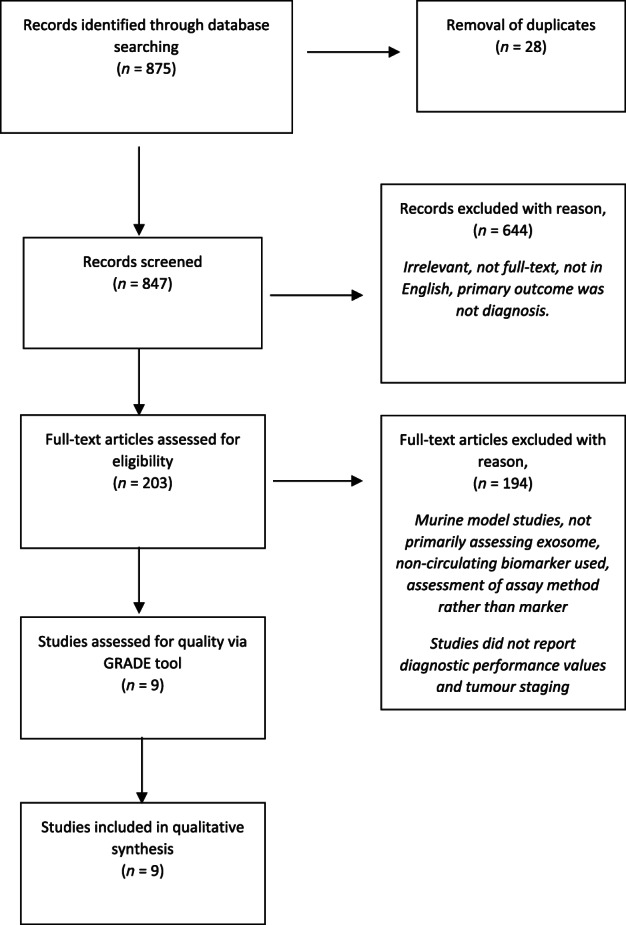
Preferred Reporting Items for Systematic Reviews and Meta‐Analyses flowchart detailing study inclusion.

The complete study inclusion process compliant with PRISMA guidelines is illustrated in Figure 2. The initial search yielded 875 articles, which included duplicates, reviews, and studies that failed to meet the inclusion criteria. Twenty‐eight duplicates were removed, and 847 records were further screened.

A total of 672 articles were excluded during screening. Reasons included articles were irrelevant, were not in English, lacked full‐text availability, or the primary focus of the study was not specifically on the diagnosis of PC.

Upon full‐text article screening, a further 194 studies were excluded. These were deemed in line with our exclusion criteria, such as assessment of murine models rather than humans, not utilizing a serum biomarker, or the study assessing an assay or spectrometry method rather than the actual biomarker in question. Some studies did not report the diagnostic performance values (sensitivity, specificity, AUC) and did not mention the tumor staging of the PC group. A total of nine articles deemed eligible for inclusion were assessed by the GRADE quality of evidence tool (Table 1) and scored “moderate.” These nine articles were used for the qualitative review. These studies were case–control studies.

**Table 1:** Quality assessment of eligible studies using the GRADE tool

Author, year, reference	Study type	GRADE score
Lewis *et al*., 2018^46^	Case–control	Moderate
Li *et al*., 2018^47^	Case–control	Moderate
Jin *et al*., 2018^45^	Case–control	Moderate
Kitagawa *et al*., 2018^41^	Case–control	Moderate
Ko *et al*., 2017^42^	Case–control	Moderate
Lai *et al*., 2017^43^	Case–control	Moderate
Melo *et al*., 2015^48^	Case–control	Moderate
Yang Z *et al*., 2020^49^	Case–control	Moderate
Wu *et al*., 2020^44^	Case–control	Moderate

Of the included studies, four assessed genetic exosomal contents^41^, ^42^, ^43^, ^44^ as biomarkers and four assessed exosomal proteins.^45^, ^46^, ^47^, ^48^ The gene arrays and proteins assessed differed between most studies (Table 2). One study subsequently used a multianalyte panel including both genetic exosomal contents and exosomal proteins.^49^ In total, the 9 studies included 331 patients and 234 controls.

**Table 2:** Summary of studies reviewed

Author, year, reference	Exosomal Biomarker(s)	Detection method	*n* (patients/controls)	Mean age	Sex (M/F)	Cancer sub‐type and pathology information	Tumor staging	Biomarker performance (Sen: %; Spe: %; AUC)	CA19‐9 performance in patient group where reported (AUC)	Comments
Lewis *et al*., 2018^46^	Panel of GPC1 and CD63	ACE immunoassay	20/11	63.7	14/6	PDAC. No histologic confirmation	T3N0: 40%, T3N1: 60%	94; 91; 0.99	—	Also distinguished PC patients from those with benign pancreatic conditions
Li *et al*., 2018^47^	MIF	SERS immunoassay	71/32	60.08	38/33	PDAC histologically diagnosed	T1–2: 10% T3: 90%	62.5; 76.2; 0.886	—	MIF distinguished P1–2 from P3–4 with a sensitivity of 95.7%
Jin *et al*., 2018^45^	ZIP4	ELISA	24/46	61.3	8/16	22 PDAC, 1 Pancreatic adenosquamous carcinoma, 1 squamous cell carcinoma of body and tail. Pathologically diagnosed	T1–2: 46% T3–4: 54%	AUC: 0.8931	—	
Kitagawa *et al*., 2018^41^	mRNAs (WASF2, ARF6) and snoRNAs (SNORA74A, SNORA25)	RT‐qPCR	27/13	60–69	17/10	PDAC pathologically diagnosed	T0–2B: 89% T3–4: 11%	AUC: >0.9 WASf2: 0.943 ARF6 0.940 SNORA74A: 0.909 SNORA25: 0.903	0.897	WASF2 mRNA may be particularly useful, as it was the most highly correlated with pancreatic cancer risk
Ko *et al*., 2017^42^	mRNA panel (ARG1, CD63, CK18, Erbb3, GAPDH, H3F3A, KRAS, ODC1)	RT‐qPCR	17/17	66	10/7	Metastatic pancreatic cancer. No histologic confirmation.	T1–2: 82% T3–4: 18%	Overall AUC: 1.0	—	
Lai *et al*., 2017^43^	microRNA panel (miR‐10b, miR‐21, miR‐30c, miR‐181a, and miR‐let7a)	RT‐qPCR	29/6	67.34	15/14	PDAC confirmed by cytopathology	T1–2: 93% T3–T4: 7%	Panel and individual biomarkers: 100; 100; 1.0	0.92 (sensitivity 86%)	Study showed decrease in exosome biomarker detection after resection of tumor
Melo *et al*., 2015^48^	GPC1	Immunogold labeling, TEM, RT‐qPCR	56/20	70	28/28	PDAC—histologically validated	T1–2: 66% T3–T4: 34%	100; 100; 1.0	0.739	Study showed decrease in biomarker post resection. Higher overall survival rate was associated in patients with lower GPC levels post resection
Yang *et al*., 2020^49^	Panel including EV‐CK18, EV‐CD63, EV‐mir‐409, ccfDNA, and CA19‐9	RT‐qPCR, ECLIA immunoassay	57/79	65.3	66/70	PDAC. No histological confirmation. Control included three IPMN	T0N0m1: 1.75% T1–2: 43.9% T3–T4: 54.4%	88,95, AUC: 0.95;	0.92	Best overall biomarker was CA19‐9. The best performing individual EV mRNA marker was CK18. The best performing EV miRNA marker was miR.409
Wu *et al*., 2020^44^	miRNA‐21, miRNA‐210	qPCR	30/10	59.1	26/14	PC confirmed by either pathology or cytology	T1–2: 20%, T3–4: 80%, ENET staging method was used. European Neuroendocrine Tumor Society	miRNA 21 80, 90, 0.869 miRNA‐210 83, 90, 0.823 Mir21/210: 93, 80 miR21/CA19‐9: 90, 90 miR‐210/CA 19–9: 90/90	0.758	The biomarkers showed higher sensitivity and specificity when combined with CA 19–9. The sensitivity and specificity of both miRNA‐21 and miRNA‐210 improved to 90% each

ACE, angiotensin convertin enzyme; AUC, area under the curve; CA19‐9, carbohydrate antigen 19‐9; PC, pancreatic cancer; PDAC, pancreatic ductal adenocarcinoma; IPMN, intraductal papillary mucinous neoplasm; MIF, macrophage migration inhibitory factor.

The median size of the study was 40 and ranged from 31 to 136 participants (Table 2). The median size of the PC population was 29.5 (range: 17–71 patients) and the median size of the control population was 18.5 (range: 6–79 controls). The male to female ratio among the patients was 1.21, and the mean age was 64.1 years. All studies included information of tumor staging to better appreciate the diagnostic utility at various stages of cancer presentation. One study included quantitative assessment of biomarker performance between early‐ and late‐stage PC.^47^ Li *et al*. showed that the discriminatory sensitivity for macrophage migration inhibitory factor (MIF) was 95.7% for early‐stage pancreatic cancer (stage 1 and 2) *versus* Stage 3 cancer.

Pathological validation of PC and its subtypes were performed in some of the included studies (Table 2). Three studies did not histologically validate the PDAC patients included.^42^, ^46^, ^49^ Lai *et al*. used cytopathology to confirm PDAC.^43^ Wu *et al*. used either cytology or pathology for validation.^44^

**Figure 3:**
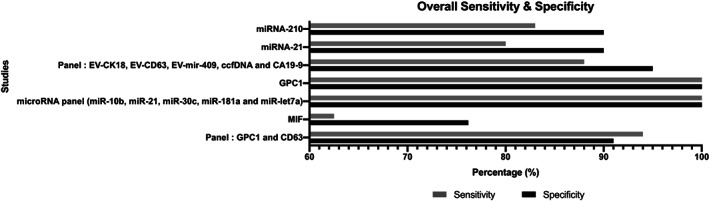
Overall reported sensitivity and specificity of exosomal biomarkers reported from studies.

Some studies also included assessment of biomarker utility in benign pancreatic conditions.^46^ Sensitivity and specificity values ranged from 62.5 to 100 and from 76.2 to 100, respectively (Fig. 3). Ko *et al*. (mRNA panel ARG1, CD63, CK18, Erbb3, GAPDH, H3F3A, KRAS, ODC1) and Melo *et al*. (GPC 1) reported the highest performing sensitivity and specificity at 100% each. Li *et al*. (MIF) reported the lowest sensitivity and specificity at 62.5% and 76.2%, respectively.

**Figure 4:**
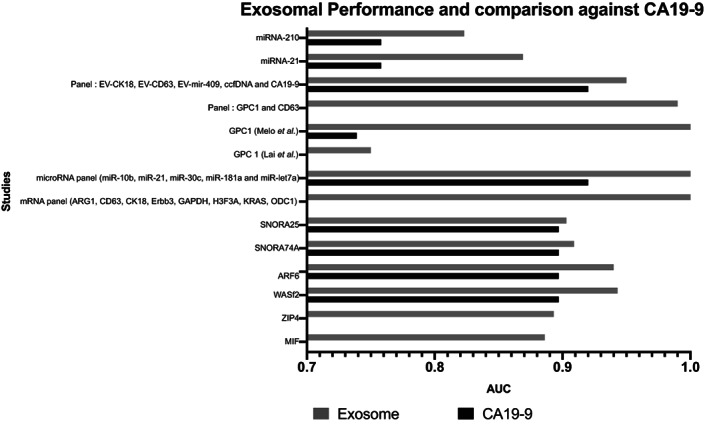
Overall area under the curve (AUC) comparison between exosomal biomarkers and CA19‐9 values (where reported) from each study. The exosomal biomarker performed better with higher AUC values in each study.

The nine studies reported the AUC values of 13 exosomal biomarkers (Table 2). The overall AUC values ranged from 0.823 to 1.0 (Fig. 4). The highest AUC performance, all showing 1.0, was reported by Lai *et al*. (microRNA panel miR‐10b, miR‐21, miR‐30c, miR‐181a, and miR‐let7a), Ko *et al*. (mRNA panel ARG1, CD63, CK18, Erbb3, GAPDH, H3F3A, KRAS, ODC1), and Melo *et al*. (GPC1). Wu *et al*. (miRNA 210) reported the lowest AUC of 0.823. Five studies^41^, ^43^, ^44^, ^48^, ^49^ compared the AUC of the exosomal biomarker against CA19‐9 (Table 2). Where reported, the AUCs of the exosomal biomarkers were superior to that of CA19‐9 (Fig. 4).

Individual biomarker discrepancy was identified between reported AUC of GPC 1 (Melo *et al*. and Lai *et al*.). Melo et al.^48^ found that it had a perfect sensitivity and specificity of 100% and that it was superior to CA19‐9 in distinguishing PC patients. Contrastingly, Lai *et al*.,^43^ found that GPC1 did not have perfect sensitivity and specificity: they determined the AUC to be 0.75 when comparing GPC1 in PC patients to normal controls. Additionally, they found that GPC1‐positive exosomes failed to adequately distinguish between PC and pancreatitis. This was further supported by Lewis *et al*.^46^ who conducted a bivariate analysis of both GPC1‐positive exosomes and CD63 exosomal proteins and reported that GPC1 did not exhibit perfect diagnostic capability.

Lai *et al*. and Wu *et al*. both assessed the performance of miRNA‐21. Sensitivity, specificity, and AUC differed (100%, 100%, and 1.0 *vs* 80%, 90%, and 0.869) between their studies.

## Discussion

A clinically reliable biomarker must demonstrate high sensitivity and specificity. In the context of PC diagnostics, it should be able to accurately discern PC from healthy control samples and it should also have a high AUC to enable detection. Establishing the accuracy of exosomal biomarkers is vital for further exploring their advantages in the earlier diagnosis of PC patients compared to existing methods.

In technical aspects, it should be able to be stored easily and not present a problem for clinicians or technicians to obtain. Also, its detection should be fairly rapid and cheap to help facilitate clinical application. It has been shown that earlier detection, in stages I/II of the classification of PC stages, improves prognosis^50^, ^51^ because often it is feasible for the patient to receive tumor resection. Furthermore, research has demonstrated that certain preneoplastic conditions precede tumor formation by nearly 8 years.^52^, ^53^ This offers a window of opportunity to pre‐emptively detect susceptibility to pancreatic tumor formation, paving the way for curative treatment and, consequently, better prognosis.

The potential of multiple exosomal biomarkers has been assessed in these studies. A key limitation identified was the differing diagnostic performances of the same biomarker between studies (GPC1 and miRNA‐21). Melo *et al*. had a larger group of PC and healthy patients, and included murine model investigations to support their findings, which therefore should not be disregarded easily. Some studies did not report the individual AUC values of the biomarkers used within the panel,^42^, ^46^ which did not allow for discrepancies to be identified for repeated biomarkers (CD63). Differences between the studies suggest that further investigation is required. Standardizing evaluations can help improve the reproducibility of results. In addition, the difference in plasma processing and immunoassay techniques can lead to differences in biomarker detection.

Various detection methods may yield different outcomes in biomarker performance. In the studies reviewed, RT‐qPCR or q‐PCR was conventionally used for miRNA and mRNA panels, and varying immunoassay methods were used for exosomal proteins (Table 2). The variation in detection methods of studies evaluating GPC1‐positive exosomes might have contributed to the differing results.^43^, ^46^, ^48^ With technological advancements, newer and more capable methods are coming to fruition. Studies should aim to assess the effectiveness for each detection method and establish which is most suitable to the detection and examination of circulating exosomes as well as other circulating biomarkers.

CA19‐9 is the most commonly used validated serum biomarker that is currently used in practice. A comparison of exosomal biomarkers that show superior performance against CA19‐9 will encourage further large‐scale studies. Not all the studies compared their exosomal biomarker's performance directly against that of CA19‐9. Nonetheless, where reported, the AUC of the exosomal biomarkers was superior to that of CA19‐9 in all the five studies. Standardization of exosomal diagnostic evaluations to include CA19‐9 comparisons will be useful in identifying the most promising biomarkers.

Where studies include different stages of PC in the patient population, biomarker performance between early‐ and late‐stage PC should be analyzed for any discrepancies. Of the reviewed study, only that of Li *et al*. reported the sensitivity, specificity, and AUC of MIF between Stage I–II *versus* Stage III PC.^47^ Such reporting would facilitate a better understanding of the accuracy of the biomarker being investigated.

It is also important to highlight the sub‐types of PC in diagnostic studies. From a histologic point of view, only a few studies clearly stated the specific sub‐types included (PDAC, adenosquamous, squamous cell, etc.) and the modality used to verify the diagnosis. Histologic verification should be performed and standardized in future studies involving PC populations.

The performance of individual exosomal biomarkers should not just be evaluated in isolation. Studies in this review that used a panel of biomarkers^42^, ^43^, ^46^ reported better outcomes compared to the performance of the biomarker in isolation. Ko *et al*.^42^ reported that only a panel of more than four genes could achieve this diagnostic accuracy, and no one single gene could solely provide such a result. They suggest that this is due to the great heterogeneity that exists among people such that several genes were differentially expressed among individuals in the group. This offers an interesting perspective of genetic biomarkers, implying that only a panel of multiple genes can be sufficient, as heterogeneity does not allow for one single gene to be significant enough to provide robust diagnosis for the wider population. The heterogenous nature of these exosomes can be of further benefit by evaluating a combination of high‐performing biomarkers.

All reviewed studies were case–control studies and had the limitation of a small sample size. Future studies should look to validate findings of these studies in larger, multicenter trials. This can extend to prospective cohort studies and examine whether earlier disease detection with the marker is translated into improved survival. This would facilitate assessing the ability of markers to predict prognosis and response to treatment.

## Conclusion

In conclusion, the potential of exosomal biomarkers remain promising in PC diagnosis, but more work needs to be done in the standardization of future studies for larger comparative analyses to overcome contrasting findings and reliably establish key pragmatic markers for clinical practice.

*Ethics statement*: This article does not contain any studies with human participants or animals performed by any of the authors.

## References

[1] Ferlay J , Colombet M , Soerjomataram I *et al*. Cancer incidence and mortality patterns in Europe: estimates for 40 countries and 25 major cancers in 2018. Eur. J. Cancer. 2018; 103: 356–87. 30100160 10.1016/j.ejca.2018.07.005

[2] Drouillard A , Manfredi S , Lepage C , Bouvier AM . Epidemiology of pancreatic cancer. Bull. Cancer. 2018; 105: 63–9. 29273548 10.1016/j.bulcan.2017.11.004

[3] He XY , Yuan YZ . Advances in pancreatic cancer research: moving towards early detection. World J. Gastroenterol. 2014; 20: 11241–8. 25170208 10.3748/wjg.v20.i32.11241 PMC4145762

[4] Becker AE , Hernandez YG , Frucht H , Lucas AL . Pancreatic ductal adenocarcinoma: risk factors, screening, and early detection. World J. Gastroenterol. 2014; 20: 11182–98. 25170203 10.3748/wjg.v20.i32.11182 PMC4145757

[5] Bilimoria KY , Bentrem DJ , Ko CY *et al*. Validation of the 6th edition AJCC Pancreatic Cancer Staging System: report from the National Cancer Database. Cancer. 2007; 110: 738–44. 17580363 10.1002/cncr.22852

[6] Hanada K , Okazaki A , Hirano N *et al*. Diagnostic strategies for early pancreatic cancer. J. Gastroenterol. 2015; 50: 147–54. 25501287 10.1007/s00535-014-1026-z

[7] Muller MF , Meyenberger C , Bertschinger P , Schaer R , Marincek B . Pancreatic tumors: evaluation with endoscopic US, CT, and MR imaging. Radiology. 1994; 190: 745–51. 8115622 10.1148/radiology.190.3.8115622

[8] Liu SL , Li S , Guo YT , Zhou YP , Zhang ZD , Lu Y . Establishment and application of an artificial intelligence diagnosis system for pancreatic cancer with a faster region‐based convolutional neural network. Chin. Med. J. 2019; 132: 2795–803. 31856050 10.1097/CM9.0000000000000544 PMC6940082

[9] Weisberg EM , Chu LC , Park S *et al*. Deep lessons learned: radiology, oncology, pathology, and computer science experts unite around artificial intelligence to strive for earlier pancreatic cancer diagnosis. Diagn. Interv. Imaging. 2020; 101: 111–5. 31629672 10.1016/j.diii.2019.09.002

[10] Chari ST . Detecting early pancreatic cancer: problems and prospects. Semin. Oncol. 2007; 34: 284–94. 17674956 10.1053/j.seminoncol.2007.05.005 PMC2680914

[11] Forsmark CE , Lambiase L , Vogel SB . Diagnosis of pancreatic cancer and prediction of unresectability using the tumor‐associated antigen CA19‐9. Pancreas. 1994; 9: 731–4. 7846016 10.1097/00006676-199411000-00010

[12] Wong D , Ko AH , Hwang J , Venook AP , Bergsland EK , Tempero MA . Serum CA19‐9 decline compared to radiographic response as a surrogate for clinical outcomes in patients with metastatic pancreatic cancer receiving chemotherapy. Pancreas. 2008; 37: 269–74. 18815548 10.1097/MPA.0b013e31816d8185

[13] Brand RE , Nolen BM , Zeh HJ *et al*. Serum biomarker panels for the detection of pancreatic cancer. Clin. Cancer Res. 2011; 17: 805–16. 21325298 10.1158/1078-0432.CCR-10-0248 PMC3075824

[14] Herreros‐Villanueva M , Gironella M , Castells A , Bujanda L . Molecular markers in pancreatic cancer diagnosis. Clin. Chim. Acta. 2013; 418: 22–9. 23305796 10.1016/j.cca.2012.12.025

[15] Chang CY , Huang SP , Chiu HM , Lee YC , Chen MF , Lin JT . Low efficacy of serum levels of CA 19‐9 in prediction of malignant diseases in asymptomatic population in Taiwan. Hepatogastroenterology. 2006; 53: 1–4. 16506366

[16] Kim JE , Lee KT , Lee JK , Paik SW , Rhee JC , Choi KW . Clinical usefulness of carbohydrate antigen 19‐9 as a screening test for pancreatic cancer in an asymptomatic population. J. Gastroenterol. Hepatol. 2004; 19: 182–6. 14731128 10.1111/j.1440-1746.2004.03219.x

[17] Goggins M . Molecular markers of early pancreatic cancer. J. Clin. Oncol. 2005; 23: 4524–31. 16002843 10.1200/JCO.2005.19.711

[18] Koopmann J , Rosenzweig CN , Zhang Z *et al*. Serum markers in patients with resectable pancreatic adenocarcinoma: macrophage inhibitory cytokine 1 versus CA19‐9. Clin. Cancer Res. 2006; 12: 442–6. 16428484 10.1158/1078-0432.CCR-05-0564

[19] Duffy MJ , Sturgeon C , Lamerz R *et al*. Tumor markers in pancreatic cancer: a European Group on Tumor Markers (EGTM) status report. Ann. Oncol. 2010; 21: 441–7. 19690057 10.1093/annonc/mdp332

[20] Rosty C , Goggins M . Early detection of pancreatic carcinoma. Hematol. Oncol. Clin. North Am. 2002; 16: 37–52. 12063828 10.1016/s0889-8588(01)00007-7

[21] Hyun KA , Kim J , Gwak H , Jung HI . Isolation and enrichment of circulating biomarkers for cancer screening, detection, and diagnostics. Analyst. 2016; 141: 382–92. 26588824 10.1039/c5an01762a

[22] Pan S , Chen R , Crispin DA *et al*. Protein alterations associated with pancreatic cancer and chronic pancreatitis found in human plasma using global quantitative proteomics profiling. J. Proteome Res. 2011; 10: 2359–76. 21443201 10.1021/pr101148r PMC3090497

[23] Pepe MS , Etzioni R , Feng Z *et al*. Phases of biomarker development for early detection of cancer. J. Natl. Cancer Inst. 2001; 93: 1054–61. 11459866 10.1093/jnci/93.14.1054

[24] Zhang X , Shi S , Zhang B , Ni Q , Yu X , Xu J . Circulating biomarkers for early diagnosis of pancreatic cancer: facts and hopes. Am. J. Cancer Res. 2018; 8: 332–53. 29636993 PMC5883088

[25] Costa‐Silva B , Aiello NM , Ocean AJ *et al*. Pancreatic cancer exosomes initiate pre‐metastatic niche formation in the liver. Nat. Cell Biol. 2015; 17: 816–26. 25985394 10.1038/ncb3169 PMC5769922

[26] Iero M , Valenti R , Huber V *et al*. Tumour‐released exosomes and their implications in cancer immunity. Cell Death Differ. 2008; 15: 80–8. 17932500 10.1038/sj.cdd.4402237

[27] Aqil F , Munagala R , Jeyabalan J , Agrawal AK , Gupta R . Exosomes for the enhanced tissue bioavailability and efficacy of curcumin. AAPS J. 2017; 19: 1691–702. 29047044 10.1208/s12248-017-0154-9

[28] Kalluri R . The biology and function of exosomes in cancer. J. Clin. Invest. 2016; 126: 1208–15. 27035812 10.1172/JCI81135 PMC4811149

[29] Sun Y , Liu J . Potential of cancer cell‐derived exosomes in clinical application: a review of recent research advances. Clin. Ther. 2014; 36: 863–72. 24863262 10.1016/j.clinthera.2014.04.018

[30] Babic A , Wolpin BM . Circulating exosomes in pancreatic cancer: will they succeed on the long, littered road to early detection marker? Clin. Chem. 2016; 62: 307–9. 26721295 10.1373/clinchem.2015.246538

[31] Tickner JA , Urquhart AJ , Stephenson SA , Richard DJ , O'Byrne KJ . Functions and therapeutic roles of exosomes in cancer. Front. Oncol. 2014; 4: 127. 24904836 10.3389/fonc.2014.00127 PMC4034415

[32] Kowal J , Arras G , Colombo M *et al*. Proteomic comparison defines novel markers to characterize heterogeneous populations of extracellular vesicle subtypes. Proc. Natl. Acad. Sci. U. S. A. 2016; 113: E968–77. 26858453 10.1073/pnas.1521230113 PMC4776515

[33] Biankin AV , Waddell N , Kassahn KS *et al*. Pancreatic cancer genomes reveal aberrations in axon guidance pathway genes. Nature. 2012; 491: 399–405. 23103869 10.1038/nature11547 PMC3530898

[34] Belczacka I , Latosinska A , Metzger J *et al*. Proteomics biomarkers for solid tumors: current status and future prospects. Mass Spectrom. Rev. 2019; 38: 49–78. 29889308 10.1002/mas.21572

[35] Batista IA , Melo SA . Exosomes and the future of immunotherapy in pancreatic cancer. Int. J. Mol. Sci. 2019; 20: 567. 30699928 10.3390/ijms20030567 PMC6387297

[36] Jiao YJ , Jin DD , Jiang F *et al*. Characterization and proteomic profiling of pancreatic cancer‐derived serum exosomes. J. Cell. Biochem. 2019; 120: 988–99. 30160795 10.1002/jcb.27465

[37] Massoumi RL , Hines OJ , Eibl G , King JC . Emerging evidence for the clinical relevance of pancreatic cancer exosomes. Pancreas. 2019; 48: 1–8. 30531240 10.1097/MPA.0000000000001203

[38] Guyatt GH , Oxman AD , Vist GE *et al*. GRADE: an emerging consensus on rating quality of evidence and strength of recommendations. BMJ. 2008; 336: 924–6. 18436948 10.1136/bmj.39489.470347.AD PMC2335261

[39] Chien PF , Khan KS , Siassakos D . Registration of systematic reviews: PROSPERO. BJOG. 2012; 119: 903–5. 22703418 10.1111/j.1471-0528.2011.03242.x

[40] Liberati A , Altman DG , Tetzlaff J *et al*. The PRISMA statement for reporting systematic reviews and meta‐analyses of studies that evaluate health care interventions: explanation and elaboration. J. Clin. Epidemiol. 2009; 62: e1–34. 19631507 10.1016/j.jclinepi.2009.06.006

[41] Kitagawa T , Taniuchi K , Tsuboi M *et al*. Circulating pancreatic cancer exosomal RNAs for detection of pancreatic cancer. Mol. Oncol. 2019; 13: 212–27. 30358104 10.1002/1878-0261.12398 PMC6360365

[42] Ko J , Bhagwat N , Yee SS *et al*. Combining machine learning and nanofluidic technology to diagnose pancreatic cancer using exosomes. ACS Nano. 2017; 11: 11182–93. 29019651 10.1021/acsnano.7b05503

[43] Lai X , Wang M , McElyea SD , Sherman S , House M , Korc M . A microRNA signature in circulating exosomes is superior to exosomal glypican‐1 levels for diagnosing pancreatic cancer. Cancer Lett. 2017; 393: 86–93. 28232049 10.1016/j.canlet.2017.02.019 PMC5386003

[44] Wu L , Zhou WB , Zhou J *et al*. Circulating exosomal microRNAs as novel potential detection biomarkers in pancreatic cancer. Oncol. Lett. 2020; 20: 1432–40. 32724386 10.3892/ol.2020.11691 PMC7377032

[45] Jin H , Liu P , Wu Y *et al*. Exosomal zinc transporter ZIP4 promotes cancer growth and is a novel diagnostic biomarker for pancreatic cancer. Cancer Sci. 2018; 109: 2946–56. 30007115 10.1111/cas.13737 PMC6125444

[46] Lewis JM , Vyas AD , Qiu Y , Messer KS , White R , Heller MJ . Integrated analysis of exosomal protein biomarkers on alternating current electrokinetic chips enables rapid detection of pancreatic cancer in patient blood. ACS Nano. 2018; 12: 3311–20. 29570265 10.1021/acsnano.7b08199

[47] Li TD , Zhang R , Chen H *et al*. An ultrasensitive polydopamine bi‐functionalized SERS immunoassay for exosome‐based diagnosis and classification of pancreatic cancer. Chem. Sci. 2018; 9: 5372–82. 30009009 10.1039/c8sc01611a PMC6009498

[48] Melo SA , Luecke LB , Kahlert C *et al*. Glypican‐1 identifies cancer exosomes and detects early pancreatic cancer. Nature. 2015; 523: 177–82. 26106858 10.1038/nature14581 PMC4825698

[49] Yang Z , LaRiviere MJ , Ko J *et al*. A multianalyte panel consisting of extracellular vesicle miRNAs and mRNAs, cfDNA, and CA19‐9 shows utility for diagnosis and staging of pancreatic ductal adenocarcinoma. Clin. Cancer Res. 2020; 26: 3248–58. 32299821 10.1158/1078-0432.CCR-19-3313 PMC7334066

[50] Poruk KE , Firpo MA , Adler DG , Mulvihill SJ . Screening for pancreatic cancer: why, how, and who? Ann. Surg. 2013; 257: 17–26. 22895395 10.1097/SLA.0b013e31825ffbfb PMC4113008

[51] Strosberg JR , Cheema A , Weber J , Han G , Coppola D , Kvols LK . Prognostic validity of a novel American Joint Committee on Cancer Staging Classification for pancreatic neuroendocrine tumors. J. Clin. Oncol. 2011; 29: 3044–9. 21709192 10.1200/JCO.2011.35.1817

[52] Brat DJ , Lillemoe KD , Yeo CJ , Warfield PB , Hruban RH . Progression of pancreatic intraductal neoplasias to infiltrating adenocarcinoma of the pancreas. Am. J. Surg. Pathol. 1998; 22: 163–9. 9500216 10.1097/00000478-199802000-00003

[53] Sugiyama M , Atomi Y . Extrapancreatic neoplasms occur with unusual frequency in patients with intraductal papillary mucinous tumors of the pancreas. Am. J. Gastroenterol. 1999; 94: 470–3. 10022648 10.1111/j.1572-0241.1999.879_h.x

